# A subject-specific mechanistic model of affect links electroencephalography, cardiorespiratory feedback, and monoaminergic dynamics

**DOI:** 10.3389/fncom.2026.1875657

**Published:** 2026-09-18

**Authors:** Masami Iwamoto

**Affiliations:** Human Science Research-Domain, Toyota Central R&D Labs., Inc., Nagakute, Aichi, Japan

**Keywords:** affect modeling, cardiorespiratory dynamics, computational neuroscience, electroencephalography, monoaminergic systems

## Abstract

**Introduction:**

Affective experience emerges from interactions among cortical activity, bodily physiology, and contextual interpretation, yet the mechanisms linking these processes remain incompletely understood. Although EEG-based studies have advanced affect decoding, many data-driven approaches provide limited mechanistic insight into inter-individual variability. We developed a subject-specific, hypothesis-generating computational framework linking EEG-derived cortical activity and cardiorespiratory dynamics to subjective affect through putative neuromodulatory processes.

**Methods:**

The framework integrates a respiratory–circulatory subsystem with spiking-neuron models representing the locus coeruleus, ventral tegmental area, raphe nucleus, and basolateral amygdala. EEG band-power features were used as hypothesis-driven, indirect inputs to these modeled processes rather than as direct measures of deep-brain activity, while cardiorespiratory variables provided physiological feedback to the neural circuit. Model-derived activity was converted into putative norepinephrine-, dopamine-, and serotonin-related signals. Together with two fitted serotonin-related modulation coefficients, these signals constituted five latent parameters used to represent valence–arousal states. The modulation coefficients were estimated for each participant–stimulus observation by nonlinear optimization so that model-derived arousal and valence matched the corresponding subjective ratings. Model calibration and predictive validation were evaluated separately. Predictive validity was assessed using leave-one-stimulus-out cross-validation, in which each stimulus was held out in turn and predicted from regression equations fitted only to the remaining 15 stimuli.

**Results:**

The framework was evaluated using the AMIGOS dataset (nine participants, 16 video stimuli; 144 participant–stimulus observations). Under the calibration procedure, the five latent parameters reproduced participant-specific valence–arousal ratings. In cross-validation across all participants, the mean root mean square error was 0.380 ± 0.288 for arousal and 1.283 ± 1.512 for valence, indicating more consistent prediction of unseen stimuli for arousal and substantial inter-individual variability in valence prediction. Cross-participant regression showed weaker generalization, further supporting the importance of subject-specific modeling.

**Discussion:**

The proposed framework does not directly measure deep-brain or monoaminergic activity. Rather, it provides a physiologically informed and interpretable framework for generating hypotheses about how cortical activity, cardiorespiratory feedback, and putative neuromodulatory processes may jointly contribute to individual affective experience. The findings also highlight the need for validation in larger independent datasets using direct neurophysiological measurements.

## 1 Introduction

Affective experience emerges from interactions between neural activity, bodily physiology, and contextual interpretation. Understanding how external stimuli shape affect therefore requires models that can bridge cortical dynamics, interoceptive signaling, and subjective experience. Although recent electroencephalography (EEG)-based studies have advanced affect decoding using data-driven and deep learning approaches, many such models remain difficult to interpret mechanistically and offer limited insight into inter-individual variability. Substantial inter-individual differences have been reported in neural and physiological responses to emotional stimuli, reflecting variability in factors such as personality, prior experience, autonomic reactivity, and cognitive appraisal (Hamman and Canli, 2004; Stemmler and Wacker, 2010). Moreover, meta-analytic evidence indicates considerable heterogeneity in autonomic nervous system responses even within nominally similar emotion categories (Siegel et al., 2018). These findings highlight the importance of subject-specific approaches that can characterize not only common physiological mechanisms but also individual patterns of brain–body coupling. Mechanistic computational modeling represents a complementary approach that connects observable signals to hypothesized neural and physiological processes, thereby enabling explicit testing of brain–body interaction hypotheses.

Monoaminergic systems are strongly implicated in affective dimensions. Norepinephrine (NE) has been associated with arousal and vigilance, dopamine (DA) with rewards-related valence, and serotonin (5-HT) with broad regulation of mood, behavioral flexibility, and decision processes (Albert et al., 2014; Lanzenberger et al., 2007). In parallel, affect is tightly coupled to autonomic and cardiorespiratory physiology, including heart rate, respiratory activity, and blood pressure (Critchley et al., 2013; Kreibig, 2010; Wu et al., 2019). Interoceptive accounts of emotion propose that bodily states are not merely consequences of emotion but actively contribute to affective experience (Barrett, 2017). Similarly, active-inference accounts emphasize the continuous interaction between central predictions and interoceptive signals in the regulation and experience of bodily states (Seth and Friston, 2016). These theoretical perspectives highlight the importance of bidirectional brain–body interactions in emotion. However, the mechanisms by which measurable cardiorespiratory variables interact with cortical and subcortical processes to shape individual valence and arousal remain incompletely understood.

Recent work in affective computing has shown that EEG and other physiological signals can be utilized to estimate affective states with increased accuracy (Galvão et al., 2021; Santamaria-Granados et al., 2019; Topic and Russo, 2021). Because existing approaches prioritize predictive performance over interpretability, they often provide limited mechanistic insight into which latent physiological processes underlie individual affective responses, why the same external stimulus may induce different affective ratings across individuals, or how cortical and bodily signals jointly contribute to these differences. These limitations are particularly relevant when the goal is not only to classify affective states but also to understand the structure of individual differences and to generate physiologically grounded hypotheses.

Other computational approaches have addressed affect from different levels of description. Reinforcement-learning models have examined how reward, prediction error, and action selection interact with affective processes (Moerland et al., 2018), whereas active-inference models emphasize inference and regulation of internal bodily states (Seth and Friston, 2016; Smith et al., 2019). These approaches provide important computational accounts of affective cognition and behavior, but the explicit integration of measured cortical activity, cardiorespiratory dynamics, and hypothesized neuromodulatory processes within a subject-specific physiological framework remains relatively unexplored.

To address this issue, a computational framework that links EEG-derived cortical activity to affect through a coupled deep-brain–cardiorespiratory system was developed. The relationships between EEG spectral features and these subcortical/brainstem regions were implemented as hypothesis-driven functional associations based on indirect neurophysiological evidence, rather than as direct physiological mappings. Building on prior cardiorespiratory modeling work (Iwamoto et al., 2024) and a limbic–locus coeruleus (LC) interaction framework (Iwamoto et al., 2023), the LC, ventral tegmental area (VTA), and raphe nucleus (RN) were modeled as spiking-neuron populations whose activities are modulated by physiological feedback variables, including the arterial partial pressure of carbon dioxide (pCO_2_), arterial partial pressure of oxygen (pO_2_), and the maximum systolic blood pressure (MAX_SBP). Their outputs were converted into NE, DA, and 5-HT signals and mapped onto the valence–arousal circumplex (Russell, 1980). In addition, two bounded serotonin modulation coefficients, *x*_1_ and *x*_2_, were introduced to represent subject- and stimulus-dependent shaping of affective state.

Using the AMIGOS dataset (Miranda-Correa et al., 2021), we examined whether this compact set of latent variables could reproduce individual valence–arousal ratings under the present calibration procedure and whether the resulting parameters could be related to EEG and cardiorespiratory variables. Rather than proposing a general-purpose affect classifier, this study aimed to present an interpretable, subject-specific, hypothesis-generating model of how cortical activity, bodily feedback, and monoaminergic dynamics may jointly shape affective experience.

The main contributions of this study are threefold. First, we introduce a subject-specific computational framework that integrates EEG-derived cortical activity with cardiorespiratory dynamics and hypothesized deep-brain neuromodulatory processes. Second, we represent affective states using a compact set of physiologically interpretable latent variables rather than relying solely on black-box classification. Third, we evaluate both goodness-of-fit and predictive validity, including within-participant generalization to unseen stimuli.

The remainder of this paper is organized as follows: section “2 Materials and methods” describes the dataset, physiological preprocessing, and computational framework. Section “3 presents the model calibration, cross-validation, and statistical analyses. Section 4 discusses the physiological interpretation, relationship to existing computational theories of emotion, potential clinical implications, and limitations. Section 5 summarizes the main conclusions and future directions.

## 2 Materials and methods

### 2.1 Model overview

A computational model that integrates (i) a cardiorespiratory system model and (ii) a limbic/deep brain circuit model was developed. The cardiorespiratory component follows our previous respiratory–circulatory framework (Iwamoto et al., 2024), and the present study extends this framework by adding limbic structures and monoaminergic nuclei based on our previous work (Iwamoto et al., 2023), as illustrated in Figure 1. The limbic circuit included the basolateral amygdala (BLA), LC, VTA, and RN, which were modeled as spiking neuron populations. The outputs were converted into monoamine signals: NE (from the LC), DA (from the VTA), and 5-HT (from the RN).

**FIGURE 1:**
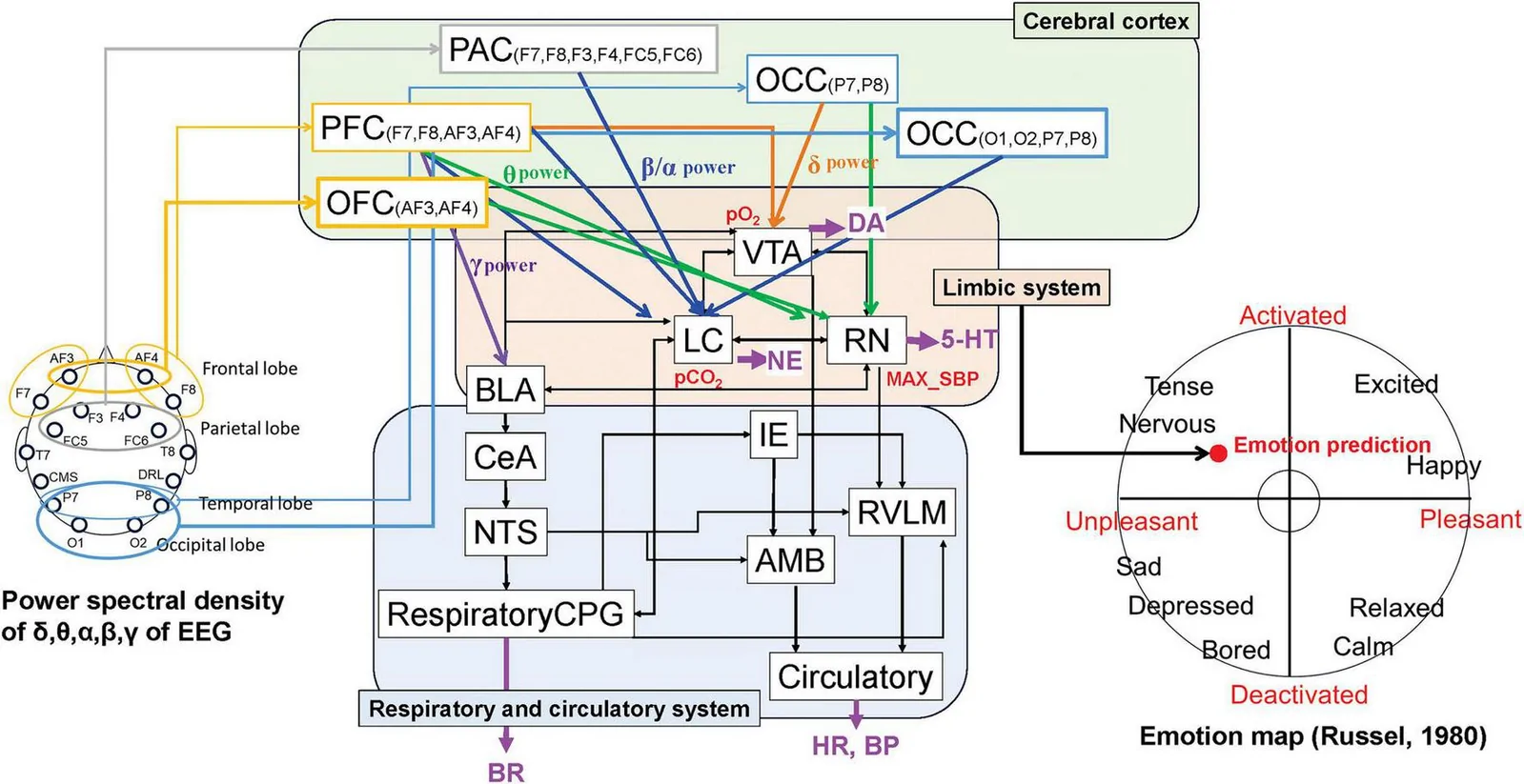
An integrated deep-brain-cardiorespiratory model calibrated to match individual valence–arousal ratings derived from electroencephalography (EEG).

### 2.2 Physiological feedback to deep-brain nuclei

To couple interoception and neuromodulation, LC activity was modulated by arterial pCO_2_, RN activity by MAX_SBP, and VTA activity by pO_2_. These feedback variables were generated by the cardiorespiratory model and fed back to the limbic/deep brain circuit during simulations.

The LC was modeled to be regulated by pCO_2_, following Quintero et al. (2017), and the RN and VTA were formulated analogously. Considering that maximal blood pressure modulates RN activity (Lindsey et al., 1998), the RN was modeled as being regulated by MAX_SBP. In addition, because the functional magnetic resonance imaging (fMRI) blood-oxygen-level-dependent (BOLD) signal in the VTA has been reported to reflect DA signaling (D’Ardenne et al., 2008), the VTA was modeled as regulated by pO_2_. Following Iwamoto et al. (2023), LC, RN, and VTA were modeled to output NE, 5-HT, and DA, respectively, as functions of their activities.

The LC model was based on Iwamoto et al. (2023) and consists of a 15 × 15 array of excitatory neurons. The activity of LC neurons was described by modifying the Hodgkin–Huxley-type equations (Ausborn et al., 2018; Shevtsova et al., 2015) as follows:


$$C\,\frac{d\,V}{d\,t}=-\varphi \,g_{L}\,\left(V-E_{L}\right)-{\bar{g}}_{K}\,m_{k}^{4}\,\left(V-E_{K}\right)$$ (1)

$$-{\bar{g}}_{N\,a}\,m_{N\,a}^{3}\,h_{N\,a}\,\left(V-E_{N\,a}\right)-I-\sqrt{2\,D}\,\xi \,\left(t\right)$$

$$-\varphi \,I_{s\,y\,n}^{E}-I_{N\,E}^{I}-I_{g\,a\,p}$$

Here, *V*denotes membrane potential and *I*denotes externally applied current. *I^E*_*syn*_is excitatory synaptic input, $I_{syn}^{E}$ is NE-mediated lateral inhibition, and $I_{NE}^{E}$ is gap-junction current described by Equation 2. The inclusion of NE-mediated inhibition was motivated by evidence that somatodendritically released NE produces α2-adrenoceptor-mediated autoinhibition and inhibition of neighboring LC neurons (Huang et al., 2012; Baral et al., 2022). Electrical coupling through gap junctions was represented using the standard ohmic formulation, in which the junctional current is proportional to the membrane-potential difference between coupled neurons (Ashwin et al., 2016; Hürkey et al., 2023):


$$I_{\text{gap}}=\underset{j}{\sum }g_{\text{gap}}\,\left(V_{i}-V_{j}\right)$$ (2)

where *g*_*gap*_ denotes the gap-junction conductance and the summation is taken over neurons electrically coupled to neuron *i* and neighboring neuron *j* contributes according to the membrane-potential difference.

The factor φ represents CO_2_-dependent modulation of ion channels and was defined using Quintero et al. (2017) as


$$\varphi =1-\frac{1}{1+{\left(s_{1/2}/s\right)}^{h_{s}}}.$$ (3)

For NE neuronal activity, *s* was assumed to depend on arterial pCO_2_ (mmHg) as


$$s=\frac{{\text{pCO}}_{2}}{760.0}\times 10^{-2},$$ (4)

following Quintero et al. (2017). To suppress the spontaneous firing rate to approximately 1 Hz, this study set *C* = 25pF and τ_*mK*_ = 1.75/*cosh*((*V* + 40)/40) ms. This study also set *g*_gap_ = 0.005 nS. The CO_2_-dependent modulation factor calculated using Equation 3 was applied to the LC neuronal dynamics in Equation 1.

Limbic–locus coeruleus neurons exerted lateral inhibition by secreting NE into the local neighborhood in response to their own excitatory firing. LC neurons release NE from somatodendritic sites, which can produce α2-adrenoceptor-mediated autoinhibition and inhibition of neighboring LC neurons (Huang et al., 2012; Baral et al., 2022). Based on this physiological evidence, spike-triggered NE release was represented using a second-order lag system, as in Iwamoto et al. (2023). Spike events were represented as delta-function inputs following standard spiking-neuron formulations (Brunel, 2000):[3-9]


$$\tau_{0}\,\dot{y}=-y+\underset{\text{j}}{\sum }\delta \,\left(t-{\text{t}}_{\text{j}}\right),$$ (5)

$$\tau_{1}\,{\dot{g}}_{\text{NE}}=-g_{\text{NE}}+y,$$ (6)

Equations 5, 6 describe the spike-triggered temporal dynamics of NE-mediated conductance, where *t_j_* denotes the occurrence time of the *j*-th spike, *g*_*NE*_ represents the normalized temporal dynamics of NE-mediated conductance following spike-triggered NE release. The resulting NE-mediated inhibitory current was then calculated using a standard conductance-based formulation (Destexhe et al., 1994), consistent with α2-adrenoceptor-mediated inhibitory conductance in computational models of LC neurons (Baral et al., 2022):


$$I_{\text{NE}}^{I}=g_{\text{NE}}^{0}\,g_{\text{NE}}\,\left(V-V_{\text{syn}}^{I}\right).$$ (7)

where $g_{\text{NE}}^{0}$ denotes the maximal NE-mediated conductance and *V^I*_*syn*_ denotes the reversal potential of the inhibitory current. In the present study, $g_{\text{NE}}^{0}=0.03\times 10^{-3}$ nS, τ_0_ = 100ms, τ_1_ = 300ms, and $V_{\text{syn}}^{I}=-75$mV. The definitions of the remaining variables follow Iwamoto et al. (2023).

Raphe nucleus and VTA neuronal activities were also described by Equation 1, but s in Equation 4 was set to (*s* = *maxBP*×0.33×(100/760.0)×10^−2^) for RN and (*s* = *pO*_2_×0.36×(100/760.0)×10^−2^) for VTA. In addition, inhibitory interactions among NE, DA, and 5-HT have been suggested previously (Guiard et al., 2008). Together with reported connections between BLA and RN (Challis and Berton, 2015; Talanov et al., 2016) and between the BLA and VTA (Averbeck and Costa, 2017; Šimić et al., 2021), these findings informed the neural circuit architecture shown in Figure 1.

### 2.3 Modeling autonomic nervous system components

As components of the autonomic nervous system, the rostral ventrolateral medulla (RVLM), a sympathetic premotor center, receives inhibitory input from the caudal ventrolateral medulla (CVLM) and exerts an excitatory effect on the myocardium. In contrast, the ambiguous nucleus (AMB), a parasympathetic premotor center, exerts an inhibitory effect on the myocardium. The RVLM, CVLM, and AMB were modeled as pacemaker neurons, following Iwamoto et al. (2023).

Based on Barnett et al. (2017), the present study introduced a population of inspiratory-to-expiratory phase-spanning neurons (IE) to provide excitatory input to the RVLM during inspiration. These IE neurons were modeled using a standard non-pacemaker spiking-neuron model. In addition, the respiratory central pattern generator (CPG) and nucleus of the solitary tract (NTS) were modeled using a standard spiking-neuron model. The NTS projects to the respiratory CPG as well as to RVLM, CVLM, and AMB and participates in respiratory and cardiac control.

In the respiratory–circulatory integrated model, NTS and the sympathetic and parasympathetic pathways regulating circulatory activity were represented using rate coding, based on the idea that neural cells encode sensory information by changes in firing rate (Hirabayashi and Iwamoto, 2019). Accordingly, the excitatory synaptic activities of NTS, RVLM, and AMB were added to the corresponding rate-coded components. To adjust the overall activity level, parameter tuning indicated that the added excitatory synaptic activities of NTS, RVLM, and AMB should be set to 100 times the difference from the previous time step.

This study also modeled the BLA, which receives input from the prefrontal cortex (PFC), using a standard spiking-neuron model. By adding BLA-to-central amygdala (CeA) input as a representation of psychological stress to the rate-coded CeA, the model was configured to emulate stress-induced changes in circulatory activity mediated by the BLA–CeA pathway (Zhang et al., 2021).

### 2.4 EEG feature extraction and cortical drive

A portion of cortical activity associated with emotional processing is reflected in scalp EEG recordings. Frontal and prefrontal oscillatory activity across multiple frequency bands has also been associated with affective processing. Among these oscillations, frontal alpha activity and, in particular, frontal alpha asymmetry have been extensively studied in relation to affective valence and approach–withdrawal motivation, whereas delta, theta, beta, and gamma activity have been associated with different aspects of emotional processing and arousal (Dixon et al., 2017; Suhaimi et al., 2020; Reznik and Allen, 2018). For example, changes in frontal alpha and delta power have been reported across positive and negative affective states in humans (Burgdorf and Moskal, 2023), and frontal theta activity has been associated with subjective emotional valence and individual differences in emotional responsiveness (Romeo and Spironelli, 2024). However, scalp EEG does not directly measure activity in deep-brain nuclei. Therefore, in the present study, the relationships between EEG spectral features and deep-brain structures were treated as hypothesis-driven functional mappings derived from converging evidence in previous electrophysiological, neuroimaging, and neurophysiological studies, rather than as direct physiological correspondences as shown in Table 1. Delta-band (δ, 0–4 Hz) activity has been associated with large-scale brain-state regulation and has been suggested to reflect neural processes involving dopaminergic circuits, although not specifically or exclusively the ventral tegmental area (Dang-Vu et al., 2005; Jaime et al., 2019). Based on these findings, delta-band power was adopted as an indirect input related to VTA-associated processing. Delta oscillations have been observed in dopaminergic neurons within the VTA (Grace, 1995), and cortical delta activity has been associated with large-scale brain-state regulation and emotional processing (Dang-Vu et al., 2005; Jaime et al., 2019; Güntekin and Başar, 2016). On the basis of this converging evidence, delta-band power was used as a biologically motivated proxy for VTA-associated processing. The β/α power ratio, defined as the ratio of beta-band (β, 13–25 Hz) power to alpha-band (α, 8–13 Hz) power, has consistently been associated with attention, vigilance, and arousal (Imagawa et al., 2023; Kahya et al., 2022). Since these cognitive functions are strongly modulated by the locus coeruleus–noradrenergic system, the β/α ratio was used as an indirect proxy for LC-related arousal regulation rather than a direct measure of LC activity. Gamma-band (γ, 25–40 Hz) oscillations play an important role in neuronal synchronization within the basolateral amygdala (Amir et al., 2018; Feng et al., 2019), and coordinated prefrontal–amygdala gamma activity has been reported during emotionally relevant social behaviors (Kuga et al., 2022). Therefore, frontal gamma-band power was hypothesized to reflect cortical processes associated with BLA function and was used as an indirect input to the BLA model. Theta-band (θ, 4–8 Hz) activity has been linked to serotonergic modulation and raphe-related neural circuits through their influence on hippocampal oscillations and prefrontal regulation (Challis and Berton, 2015; Jelitai et al., 2021; Sörman et al., 2011). Accordingly, theta-band power was treated as a functional indicator related to RN-associated processing rather than a direct measure of raphe nucleus activity. Throughout this study, these EEG-to-nucleus mappings should therefore be interpreted as biologically informed computational hypotheses introduced to construct an interpretable neuromodulatory framework. Their physiological validity should be further examined in future studies using multimodal approaches, such as simultaneous EEG-fMRI, PET imaging, or invasive neural recordings. Accordingly, in the present model, δ, β/α, γ, and θ power values were applied as bias current inputs to VTA, LC, BLA, and RN, respectively.

**TABLE 1:** Hypothesized functional relationships between electroencephalography (EEG) features and deep-brain regions.

EEG feature	Hypothesized functional correlate	Supporting evidence	Evidence level
δ power	VTA-associated processing	Dang-Vu; Jaime; Guntekin; Grace	Indirect
β/α ratio	LC-related arousal regulation	Imagawa; Kahya	Indirect
γ power	BLA-associated processing	Amir; Feng; Kuga	Indirect
θ power	RN-associated processing	Challis; Jelitai; Sorman	Indirect

These relationships represent biologically informed hypotheses based on indirect evidence and should not be interpreted as direct physiological mappings between scalp EEG activity and deep-brain nuclei.

To account for inter-individual variability, personality-related differences in EEG feature selection were also considered. Specifically, this study referred to the HEXACO model (Ashton et al., 2004), which characterizes personality in terms of honesty–humility, emotionality, extraversion, agreeableness, conscientiousness, and openness. Prior studies suggest that higher agentic extraversion is associated with lower θ-band power in the orbitofrontal cortex (OFC) and higher δ- and θ-band activity in occipital regions, including the posterior cingulate cortex and precuneus (Knyazev et al., 2012). Higher agreeableness has been associated with lower α-band activity and higher β-band activity in parietal and occipital regions (Jach et al., 2020). Honesty–humility has also been related to empathy, and activity in the temporoparietal junction (TPJ) has been linked to empathy and altruism (Karpouzian-Rogers et al., 2021; Morishima et al., 2012; Zebarjadi et al., 2023). Based on these findings, α-band power was extracted from parietal and frontal regions including the TPJ. More broadly, frontal and fronto-posterior EEG spectral patterns have been associated with personality traits. For example, agentic extraversion has been associated with lower theta activity in the orbitofrontal cortex (Knyazev et al., 2012), while multivariate resting-state EEG power has shown preliminary associations with Big Five personality traits (Jach et al., 2020). These findings motivate consideration of personality-related individual differences in EEG features, although they do not imply a one-to-one correspondence between a specific frequency band and a specific personality trait. The final EEG extraction sites and frequency bands are summarized in Table 2.

**TABLE 2:** Electroencephalography (EEG) recording sites and frequency bands used to compute power values from the EEG data.

Name of EEG power	Brain regions	EEG electrode placement
δ (PFC)	Prefrontal/orbitofrontal cortex	F7, F8, AF3, AF4
θ (PFC)	Prefrontal/orbitofrontal cortex	F7, F8, AF3, AF4
α (PFC)	Prefrontal/orbitofrontal cortex	F7, F8, AF3, AF4
β (PFC)	Prefrontal/orbitofrontal cortex	F7, F8, AF3, AF4
γ (PFC)	Prefrontal/orbitofrontal cortex	F7, F8, AF3, AF4
δ (OCC)	Posterior cingulate cortex, precuneus	P7, P8
θ (OCC)	Posterior cingulate cortex, precuneus	P7, P8
α (OCC)	Posterior cingulate cortex, precuneus, occipital cortex	O1, O2, P7, P8,
β (PAC)	Parietal cortex	F3, F4, FC5, FC6
θ (OFC)	Orbitofrontal cortex	AF3, AF4
α (PFC/PAC)	Prefrontal/parietal cortex	F7, F8, F3, F4, FC5, FC6

### 2.5 Calculation of arousal and valence from EEG power measures

The 11 EEG power measures listed in Table 2 were treated as current values reflecting cortical activity in the PFC, posterior cingulate cortex/precuneus/occipital cortex (OCC), parietal cortex (PAC), and OFC. These currents were then provided as inputs to the limbic system model based on prior studies linking EEG power features to BLA, LC, VTA, and RN (Amir et al., 2018; Challis and Berton, 2015; Dang-Vu et al., 2005; Feng et al., 2019; Imagawa et al., 2023; Jaime et al., 2019; Jelitai et al., 2021; Kahya et al., 2022; Kuga et al., 2022; Sörman et al., 2011). As shown in Figure 1, activity in the limbic system model is conveyed to the NTS, a central node of respiratory–circulatory regulation. The resulting changes in pCO_2_, pO_2_, and MAX_SBP were fed back to LC, VTA, and RN, respectively. Using the model-derived activities of LC, VTA, and RN over a predefined time window, NE, DA, and 5-HT were computed.

The present study defined affective state represented on the circumplex model of affect (Russell, 1980) using the following equations:


$$\text{Arousal}=\left[\text{NE}\right]-x_{1}\,\left[5\,\text{-}\,\mathrm{HT}\right],$$ (8)

$$\text{Valence}=\left[\text{DA}\right]+x_{2}\,\left[5\,\text{-}\,\mathrm{HT}\right],$$ (9)

$$\left|{\text{x}}_{1}\right|<1.5,\left|{\text{x}}_{2}\right|<1.5.$$ (10)

Here, the bracketed quantities represent the difference between the mean values of NE, DA, and 5-HT over the 70-s interval from 20 to 90 s after simulation onset and the corresponding mean values over the 10-s pre-stimulus interval from 10 to 20 s. Because the effects of serotonin on arousal and valence are not fully established and may vary depending on context (Monti, 2011; Blier, 2001; Harkin et al., 2025), *x*_1_ and *x*_2_ were allowed to take either positive or negative values within bounded ranges specified in Equation 10. For each participant–stimulus sample, *x*_1_ and *x*_2_ were identified so that the model outputs matched the self-assessed arousal and valence ratings.

### 2.6 Application of the model to the AMIGOS dataset

The model was applied to the AMIGOS dataset (Miranda-Correa et al., 2021), which provides publicly available EEG, electrocardiogram (ECG), galvanic skin response (GSR), and self-assessment data for affective stimulation experiments. The dataset includes 16 short movie clips intended to evoke affective states distributed across the four quadrants of the circumplex model: high valence/high arousal (HVHA), low valence/high arousal (LVHA), low valence/low arousal (LVLA), and high valence/low arousal (HVLA). Although 10 participants consented to the use of their EEG data, 1 participant (No. 33) had missing EEG recordings for 10 of the 16 short-video conditions. Therefore, the present analyses included nine participants.

During viewing, 14-channel EEG, ECG, and GSR were recorded, and each participant provided 9-point Self-Assessment Manikin (SAM) ratings of valence, arousal, and dominance. The EEG electrodes follow the international 10–20 system and correspond to the Emotiv EPOC Neuroheadset used in AMIGOS.

From the raw 14-channel EEG recordings (0–50 s), power spectral density (PSD) features were extracted. Signals were sampled at 128 Hz, average-referenced, and high-pass filtered at 2 Hz. Ocular artifacts were removed using blind source separation (Gomez-Herrero et al., 2010). PSD was estimated using Welch’s method with a 2-s Hamming window and 50% overlap. For each of the 11 frequency-band definitions listed in Table 2, band power was computed by trapezoidal integration, and the corresponding δ, βα, γ, and θ values were used as bias inputs to VTA, LC, BLA, and RN, respectively.

### 2.7 Simulation protocol

Figure 2 illustrates the simulation workflow. Although clip duration varied across movies, this study used the first 50 s of data after stimulus onset and computed the EEG power measures listed in Table 2. For each participant and each movie stimulus, these EEG power features were provided as inputs to the model.

**FIGURE 2:**
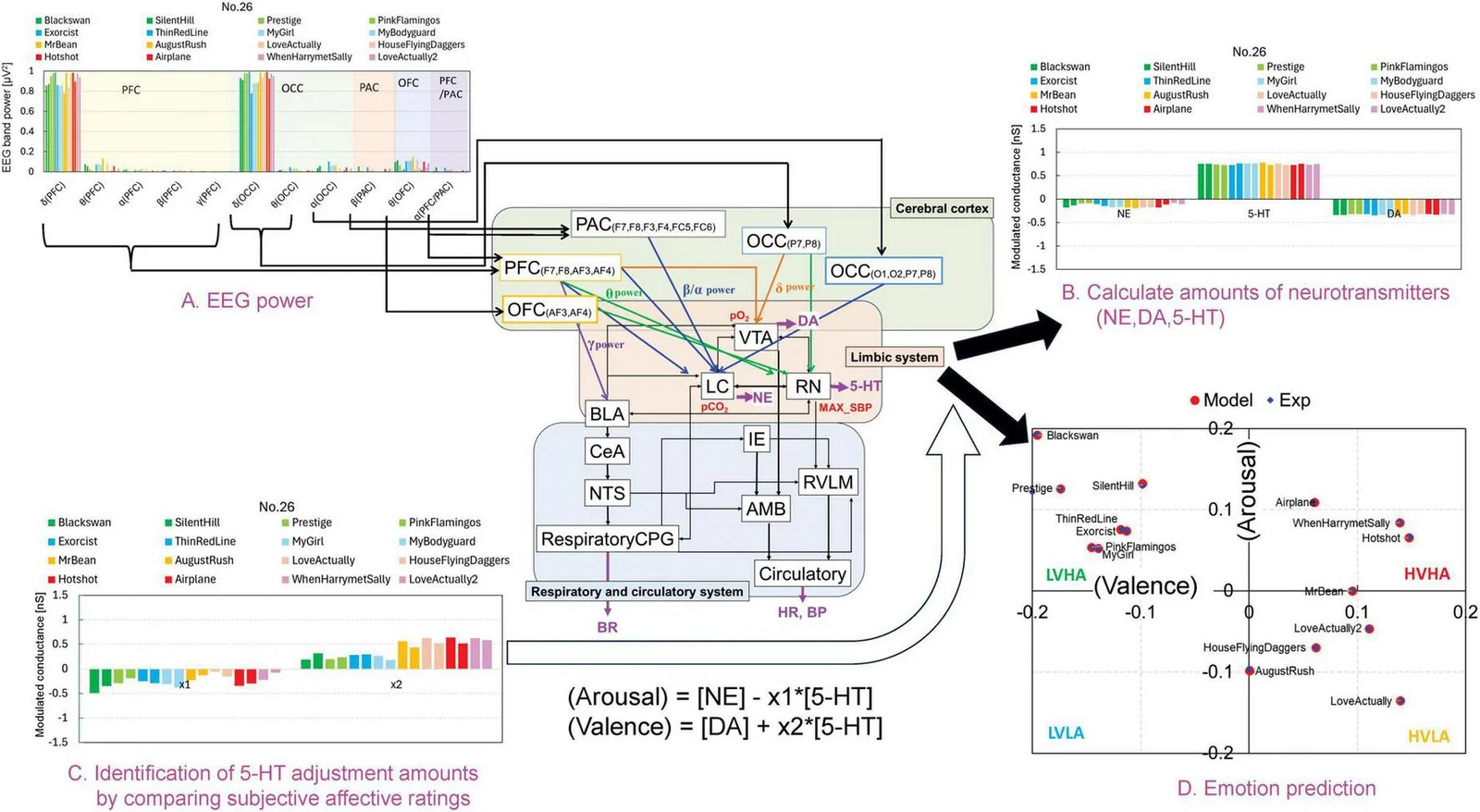
Workflow of model-based affect prediction (example: Participant No. 26). **(A)** Input of EEG power values; **(B)** computation of neurotransmitters (NE, DA, and 5-HT); **(C)** identification of 5-HT adjustment terms that fit the subjective ratings; and **(D)** comparison between predicted affect and subjective ratings.

The first 20 s of simulation were run without stimulus input to stabilize the respiratory–circulatory subsystem (Iwamoto et al., 2024). Subsequently, for a 50-s interval, the EEG power values at each site were held constant and applied as inputs to the limbic system. Following this stimulation period, an additional 20 s was simulated without stimulus input, yielding a total simulation time of 90 s.

For pCO_2_, pO_2_, and MAX_SBP, feedback quantities were computed as the differences between the maximum values over the 70-s interval from 20 to 90 s and the maximum values over the 10-s pre-stimulus interval from 10 to 20 s (38.4 mmHg, 86.6 mmHg, and 111.1 mmHg, respectively). Mean values of NE, DA, and 5-HT over the 70-s interval from 20 to 90 s were then computed. Following Equations 7, 8 and using the GRG Nonlinear algorithm in the Solver add-in of Microsoft Excel, this study identified *x_1_* and *x_2_* such that the resulting arousal and valence matched the subjective ratings.

As an auxiliary validation of the cardiorespiratory component, the PSD of heart rate variability (HRV) over the 90-s simulation was also computed and compared with the HRV PSD derived from the AMIGOS ECG data. HRV PSD was computed using pyHRV (Gomes et al., 2019), an open-source Python toolbox. Initially, the simulated HRV exhibited a larger high-frequency component than the very-low-frequency component, unlike the AMIGOS data. This discrepancy was attributable to the parameter *k*_*resp*_, which determines the slope of the sigmoid function in the parasympathetic firing-rate equation (Hirabayashi and Iwamoto, 2019). To better match the experimental HRV PSD pattern, *k*_*resp*_ was changed from 0.08 to 2.0 (Iwamoto et al., 2024).

### 2.8 Statistical analyses

Multiple regression analyses were performed to relate the predicted arousal and valence (which matched the subjective ratings by construction) to 14 physiological variables (11 EEG power features + three cardiorespiratory variables: pCO_2_, pO_2_, MAX_SBP). The model fit was assessed using R^2^ and adjusted R^2^ values. For Within-participant generalization, the regression models included 13 physiological predictors (11 EEG power variables, pCO_2_, and pO_2_). The number of predictors was restricted to 13 because inclusion of all 14 candidate variables resulted in unstable coefficient estimation given the limited number of observations available within each participant. The Lindeman–Merenda–Gold (LMG; Shapley-based) and Pratt indices were used to quantify variable importance and contribution signs (Draper and Smith, 1998; Kutner et al., 2005). LMG and Pratt importance metrics were computed using custom Excel macros developed with assistance from ChatGPT. Moreover, cross-participant versus within-participant generalizations were assessed using participant-specific regression models.

## 3 Results

### 3.1 Five latent parameters reproduced individual valence–arousal ratings

Figure 3 presents the comparison of model-derived affective states and subjective ratings for Participants No. 25, No. 26, No. 30, and No. 31. Red circles indicate model-based affect estimates, and blue diamond’s indicate the experimentally obtained self-assessment values. While data from four representative participants are shown in Figure 3, the model yielded close agreement with the subjective ratings for all 16 video conditions in all nine participants. The corresponding results for Participants No. 09, No. 37, No. 38, No. 39, and No. 40 are shown in Supplementary Figure 1.

**FIGURE 3:**
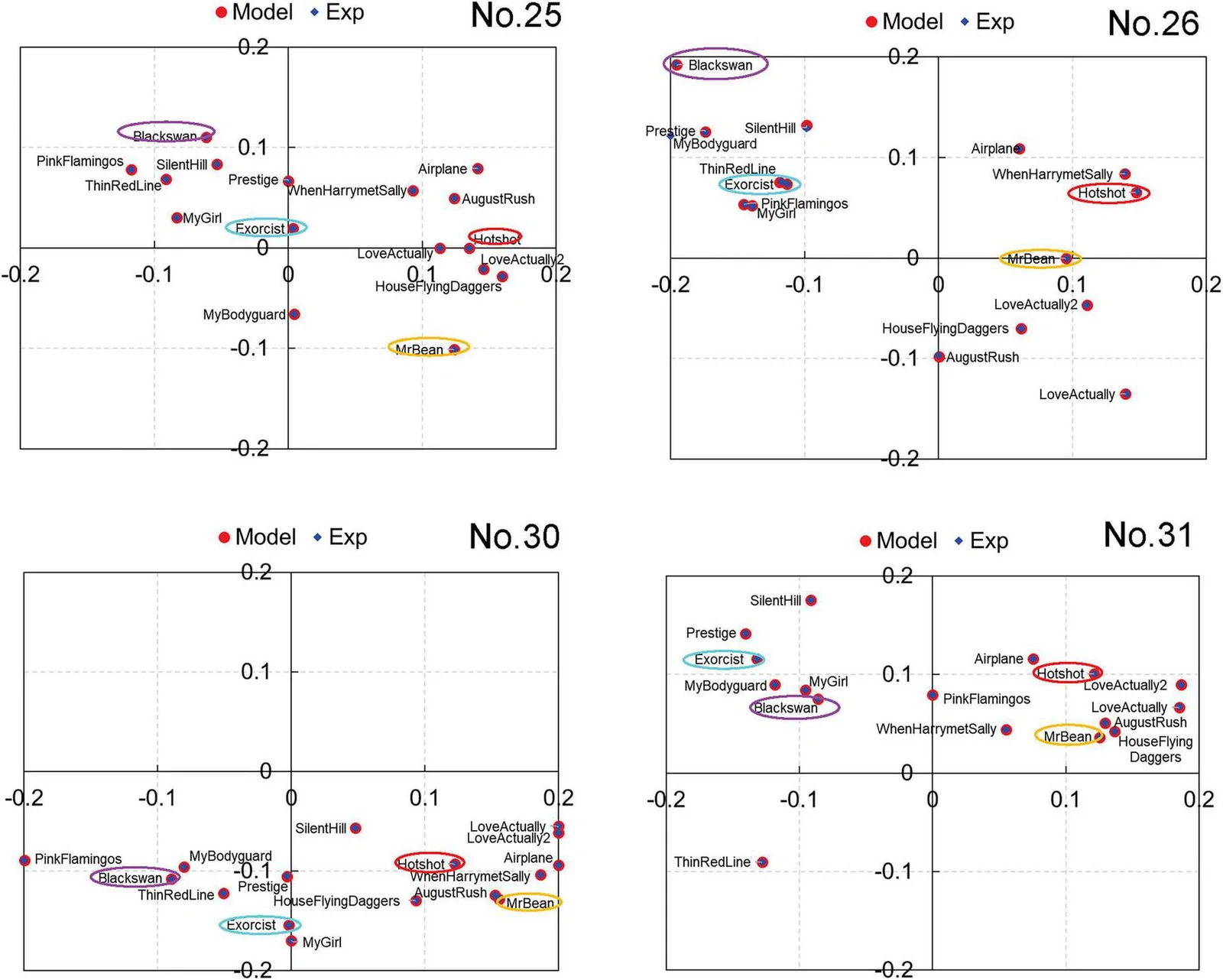
Comparison between model-predicted affect and subjective ratings (Participants No. 25, No. 26, No. 30, and No. 31). In each plot, the x-axis represents valence and the y-axis represents arousal. Text labels indicate the titles of the 16 movies.

To illustrate inter-individual differences in subjective evaluation, four representative movies corresponding to the four quadrants of the circumplex model were selected: *Hotshot* (HVHA), *Blackswan* (LVHA), *Exorcist* (LVLA), and *MrBean* (HVLA). Even for the same movie, participants sometimes rated the stimulus in different quadrants. For example, *Exorcist* was expected to evoke an LVLA state, yet Participant No. 25 rated it in the first quadrant, whereas Participants No. 26 and No. 31 rated it in the second quadrant. Participant No. 30 rated all 16 movies in the third and fourth quadrants, indicating consistently low arousal, whereas Participant No. 31 rated 15 of the 16 movies in the first and second quadrants, indicating consistently high arousal. These indicate that the model was able to accommodate marked inter-participant differences in affective ratings through the five parameters NE, DA, 5-HT, *x*_1_, and *x*_2_.

For Participant No. 26, the estimated values of NE, DA, 5-HT, *x*_1_, and *x*_2_ for the 16 video stimuli, together with the subjective arousal and valence ratings, are listed in Table 3. NE, DA, and 5-HT were defined as changes from the pre-stimulus baseline. *x*_1_ and *x*_2_ were identified so that the model outputs matched the subjective ratings under Equations 8, 9. The corresponding results for the remaining participants are provided in Supplementary Tables 1–4.

**TABLE 3:** Predicted values of norepinephrine (NE), dopamine (DA), serotonin (5-HT), *x*_1_, and *x*_2_, and the subjective arousal and valence ratings for the 16 video stimuli (Participant No. 26).

Video names	Short form	NE	DA	5-HT	x1	x2	Arousal (SAR)	Valence (SAR)
Black Swan	Blackswan	−0.179	−0.337	0.758	−0.490	0.186	0.193	−0.196
Silent Hill	SilentHill	−0.133	−0.335	0.756	−0.351	0.313	0.130	−0.099
Prestige	Prestige	−0.090	−0.320	0.740	−0.292	0.196	0.126	−0.174
Pink Flamingos	PinkFlamingos	−0.083	−0.318	0.728	−0.188	0.237	0.054	−0.145
The Exorcist	Exorcist	−0.109	−0.320	0.728	−0.251	0.284	0.074	−0.113
The Thin Red Line	ThinRedLine	−0.145	−0.344	0.763	−0.289	0.295	0.077	−0.118
My Girl	MyGirl	−0.182	−0.340	0.763	−0.307	0.262	0.052	−0.139
My Bodyguard	MyBodyguard	−0.167	−0.340	0.762	−0.380	0.183	0.122	−0.200
Mr. Beans’ Holiday	MrBean	−0.180	−0.344	0.783	−0.230	0.561	0.000	0.096
August Rush	AugustRush	−0.193	−0.318	0.728	−0.130	0.437	−0.097	0.000
Love Actually (DECAF)	LoveActually	−0.177	−0.335	0.763	−0.056	0.622	−0.135	0.141
House of Flying Daggers	HouseFlyingDaggers	−0.182	−0.320	0.729	−0.154	0.523	−0.070	0.062
Hot Shots	Hotshot	−0.182	−0.318	0.728	−0.340	0.640	0.066	0.149
Airplane	Airplane	−0.115	−0.330	0.756	−0.296	0.516	0.109	0.061
When Harry Met Sally	WhenHarrymetSally	−0.079	−0.318	0.732	−0.223	0.625	0.085	0.139
Love Actually (MAHNOB-HCI)	LoveActually2	−0.103	−0.322	0.746	−0.076	0.581	−0.047	0.111

SAR, subjective affective rating.

### 3.2 Within-participant generalization was relatively strong, whereas cross-participant generalization was weak

Multiple regression analyses were performed between the affective ratings and 14 physiological variables: 11 EEG power measures and three cardiorespiratory measures (pCO_2_, pO_2_, and MAX_SBP). Figure 4 shows the results for Participants No. 26 and No. 30. The x-axis represents the model-derived affective values, which matched the subjective ratings under the present calibration procedure, and the y-axis represents the values predicted by the regression equations using the 14 physiological variables. For both participants, the coefficients of determination for arousal and valence exceeded 0.9.

**FIGURE 4:**
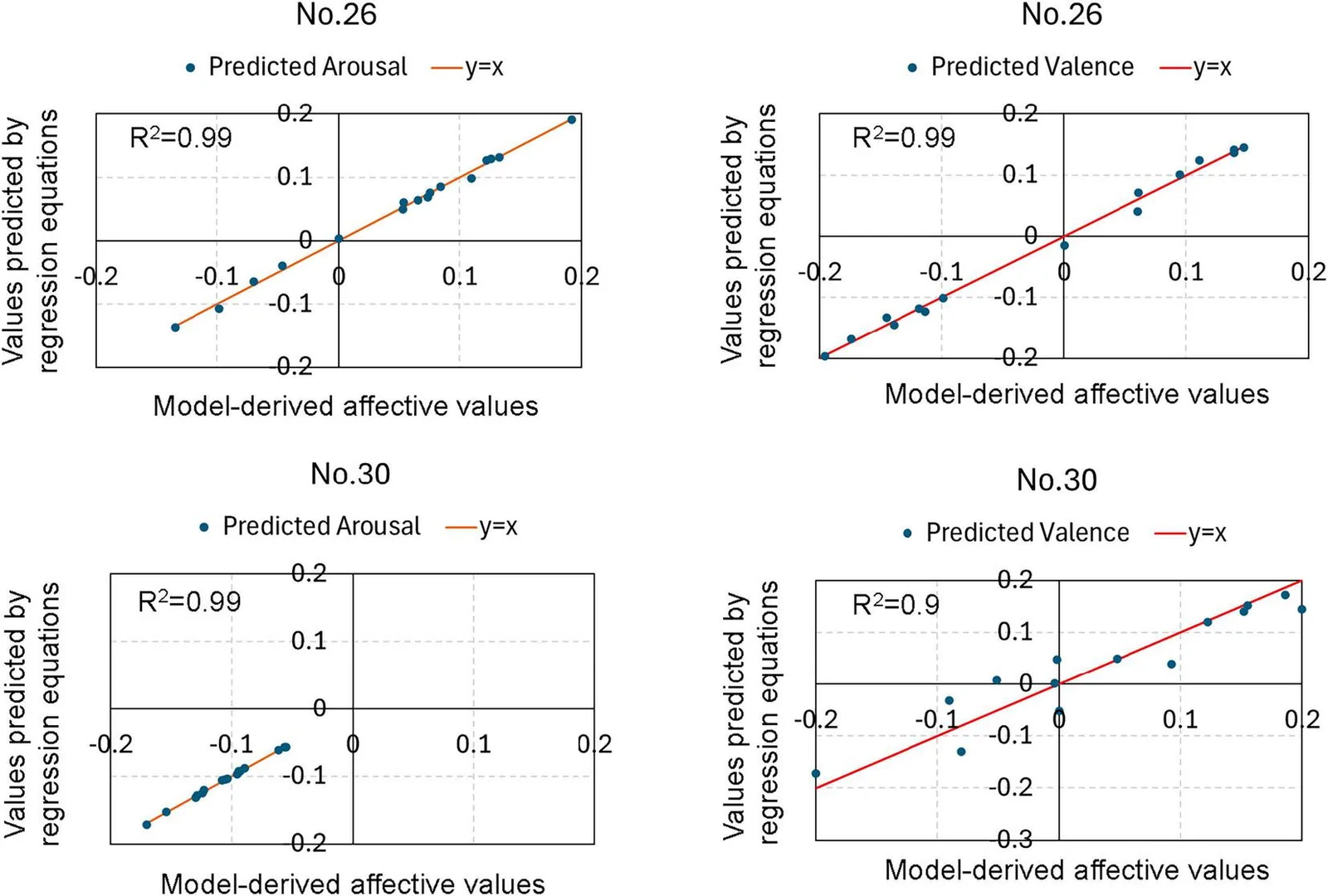
Relationship between model-derived affect estimates (=subjective ratings) and fitted values from the multiple regression model using 14 variables (Participants No. 26 and No. 30).

Table 4 summarizes the R^2^ values for arousal and valence for each participant and for the pooled dataset. Results obtained using only the 11 EEG power variables, excluding the three cardiorespiratory measures, are also reported. Excluding the cardiorespiratory variables substantially reduced R^2^, indicating that these physiological signals contributed importantly to the model-derived affective structure. However, although participant-specific regression models showed high fit, a single model fitted across all participants yielded poor fit (R^2^ ≈0.1), suggesting marked inter-individual variability.

**TABLE 4:** *R*^2^ values for arousal and valence using 14 variables and 11 variables (for each of the nine participants and for the pooled data across all nine participants).

Participant no.	Arousal (14 variables)	Valence (14 variables)	Arousal (11 variables)	Valence (11 variables)
09	**0.95**	0.84	**0.90**	0.64
25	**0.94**	**0.95**	0.88	0.85
26	**0.99**	**0.99**	**0.93**	0.88
30	**0.99**	**0.90**	0.73	0.53
31	**0.97**	**0.99**	0.87	**0.94**
37	**0.97**	**0.99**	**0.97**	0.89
38	**0.96**	**0.97**	0.74	0.64
39	**0.94**	0.76	**0.90**	0.40
40	**0.99**	**0.98**	0.88	0.54
All	0.14	0.10	0.11	0.09

Cases with *R*^2^ ≥ 0.9 are shown in bold.

To assess generalization, Participant No. 26, whose regression models showed the highest R^2^, was assessed as a reference case. For cross-participant generalization, the regression equations of this participant were applied to the data of others, and the signs of predicted and observed arousal and valence were compared. A sample was considered “reproduced” when both signs agreed. As shown in Table 5, only three participants for arousal and one participant for valence achieved reproduction rates of 80% or higher, indicating weak cross-participant generalization.

**TABLE 5:** Reproduction rates of subjective ratings when applying the regression equations of Participant No. 26 (using 14 variables) for arousal and valence to other participants.

Participant no.	Arousal (%)	Valence (%)
09	63	56
25	**81**	31
30	**94**	50
31	44	44
37	44	56
38	**88**	69
39	50	63
40	38	**81**

Cases with reproduction rates ≥ 80% are shown in bold.

To evaluate the predictive validity of the model for unseen stimuli, leave-one-stimulus-out (LOSO) cross-validation was performed separately for all nine participants. For each participant, one of the 16 movie conditions was held out as the test condition, while regression models for arousal and valence were fitted using the remaining 15 conditions. The regression models included 13 physiological predictors comprising 11 EEG power variables and two cardiorespiratory variables (pCO_2_ and pO_2_). The fitted regression equations were then applied to the physiological variables of the held-out condition to predict its arousal and valence ratings. This procedure was repeated 16 times for each participant so that each movie condition served once as an unseen test condition. Importantly, data from the held-out condition were not used to estimate the regression coefficients.

Prediction performance was quantified using the root mean square error (RMSE) between the predicted and observed ratings across the 16 held-out conditions for each participant. The participant-specific RMSE values are summarized in Table 6, and their distributions across participants are shown in Figure 5. Across the nine participants, the mean RMSE was 0.380 ± 0.288 for arousal and 1.283 ± 1.512 for valence. The larger mean RMSE and inter-participant variability observed for valence indicate that the model generalized more consistently to unseen stimuli for arousal than for valence.

**TABLE 6:** Prediction errors obtained from leave-one-stimulus-out cross-validation for each participant.

Participant no.	RMSE (arousal)	RMSE (valence)
09	0.598	0.560
25	0.334	0.512
26	**0.151**	**0.266**
30	**0.250**	2.380
31	**0.120**	**0.190**
37	1.021	4.917
38	0.490	0.770
39	**0.280**	1.179
40	**0.174**	0.776
Mean ± SD	0.380 ± 0.288	1.283 ± 1.512

Cases with RMSE ≤ 0.3 are shown in bold.

**FIGURE 5:**
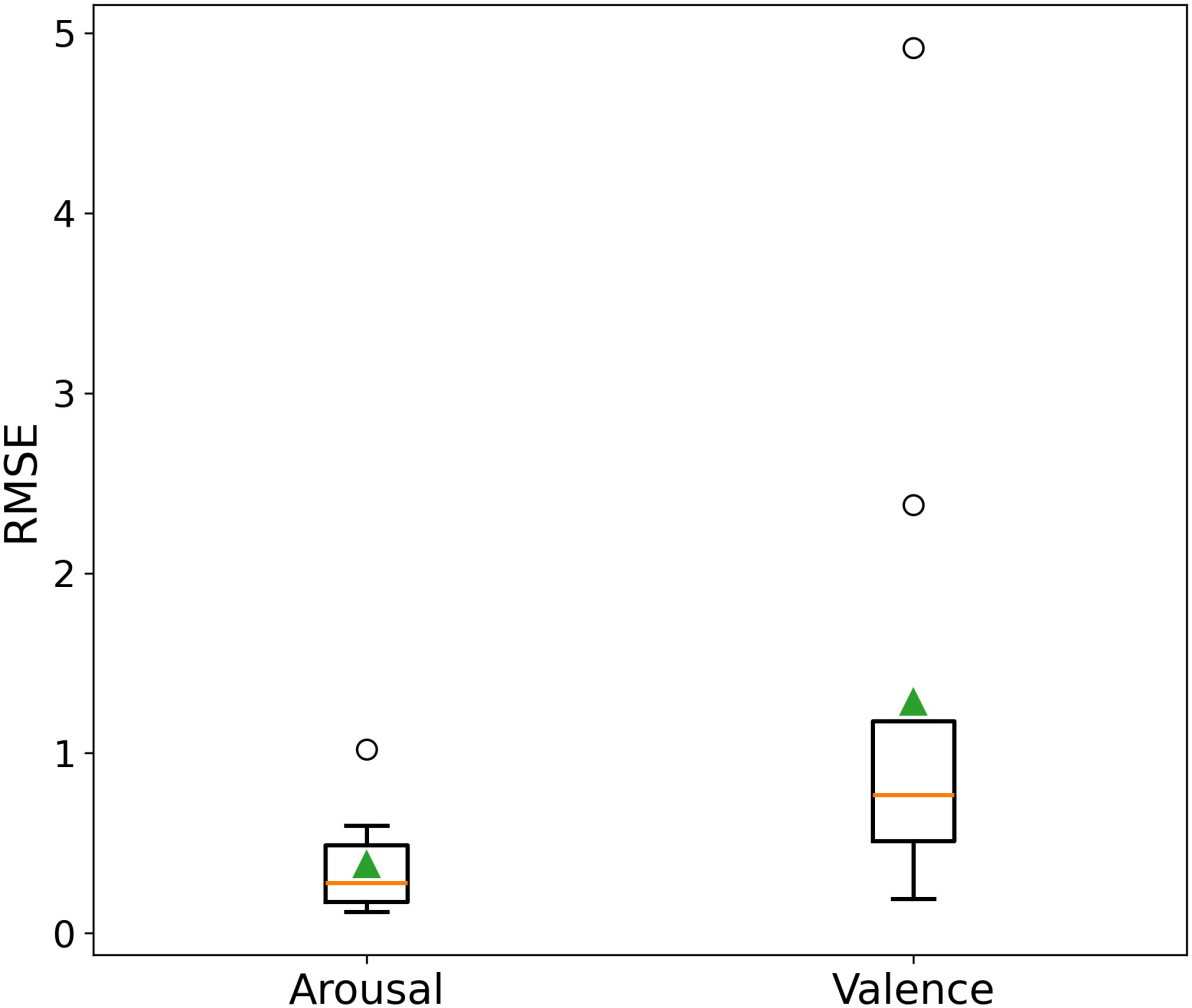
Distribution of prediction errors across participants in leave-one-stimulus-out cross-validation. Boxplots show the distributions of participant-specific RMSE values for arousal and valence. Triangles indicate the mean values, and circles indicate outliers.

These cross-validation results evaluate predictive performance on held-out stimuli and should be distinguished from the goodness-of-fit measures reported in Figure 3, which quantify the reproduction of ratings using fitted model parameters.

### 3.3 Serotonin modulation expanded affective states across the circumplex

Figure 6 shows the predicted affective states for all nine participants before and after serotonin adjustment. The rectangular region on the left side of the figure corresponds to the case *x*_1_ = *x*_2_ = 0, i.e., the state before serotonin adjustment.

**FIGURE 6:**
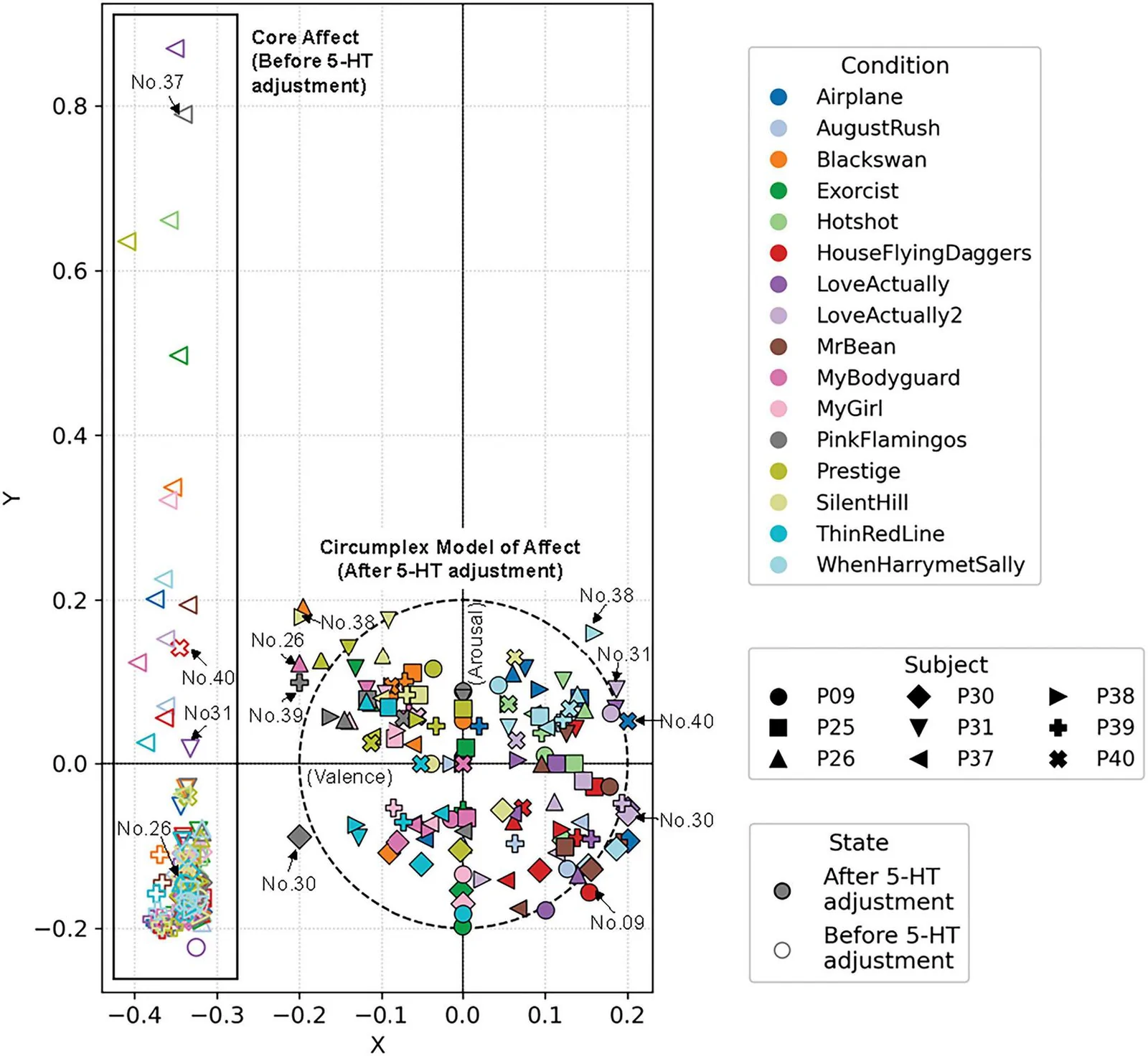
Predicted affect before and after 5-HT adjustment (results for all nine participants). The x-axis represents valence and the y-axis represents arousal. Points with different colors and shapes indicate affect predictions for different participants. Points inside the rectangle on the left indicate predictions before 5-HT adjustment, whereas points on and around the circumplex circle indicate predictions after 5-HT adjustment.

Before serotonin adjustment, the predicted states were distributed mainly in the second and third quadrants of the circumplex, corresponding to unpleasant affective states. Specifically, second-quadrant predictions occurred for 15 stimulus conditions in Participant No. 37 and for one stimulus condition each in Participants No. 31 and No. 40; all remaining predictions fell into the third quadrant. In contrast, after serotonin adjustment, the predicted states extended across the circumplex for almost all participants and stimuli. A small number of conditions lay slightly outside the circumplex range because the corresponding subjective ratings themselves fell outside the nominal circumplex boundary.

Figure 7 shows the estimated quantities of the three monoamines and the two serotonin modulation terms for Participants No. 26, No. 31, and No. 37 across the 16 stimulus conditions. Results for the other participants are shown in Supplementary Figures 2, 3. Among the three monoamines, DA and 5-HT varied only modestly across stimuli, whereas NE showed somewhat greater stimulus dependence. In contrast, both *x*_1_ and *x*_2_ varied strongly across stimulus conditions. These findings suggest that NE and serotonin-related modulation were more sensitive to external stimuli than DA and 5-HT.

**FIGURE 7:**
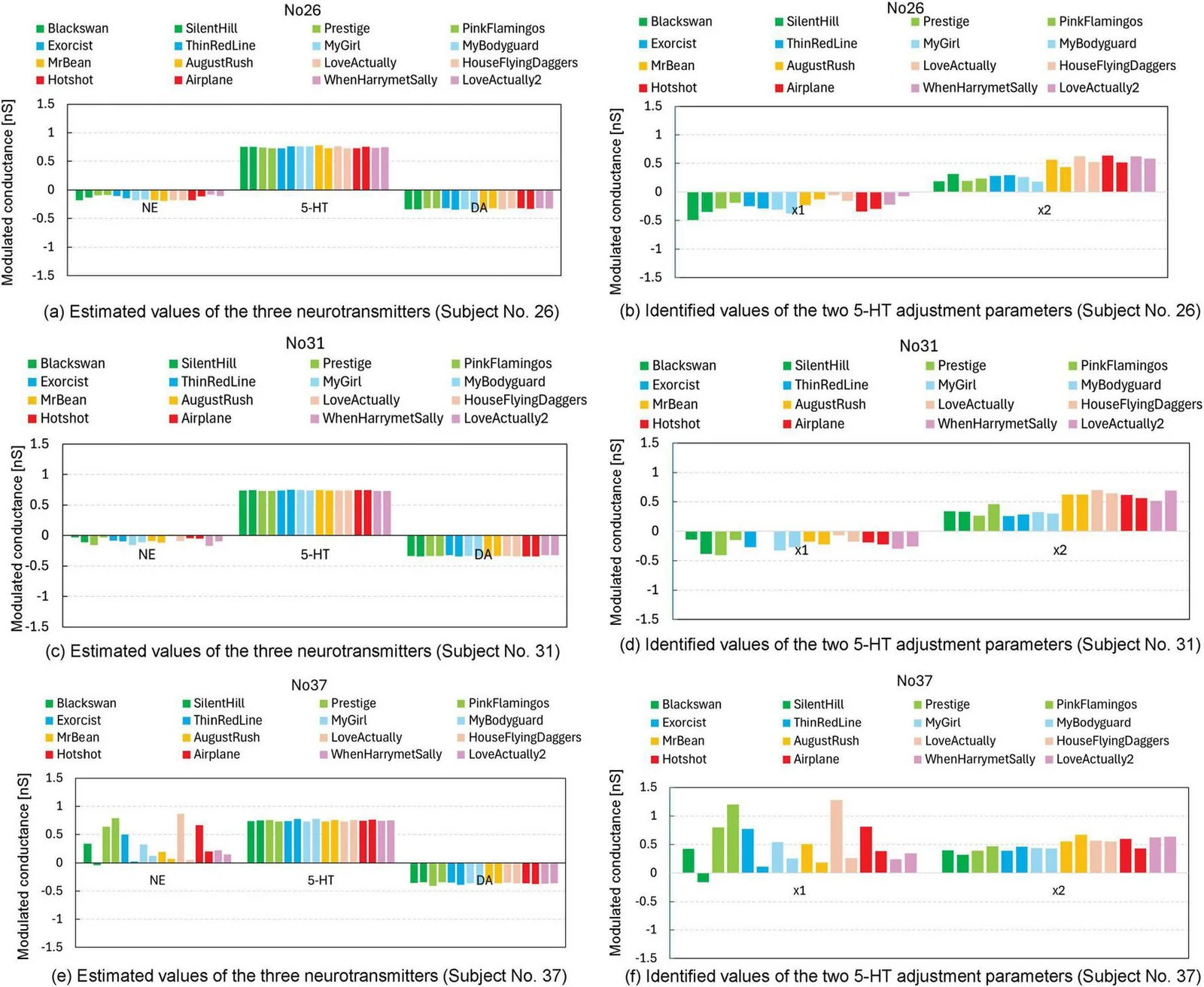
Computed quantities of the three neurotransmitters and the two 5-HT adjustment terms across the 16 video stimulus conditions (Participants No. 26, No. 31, and No. 37).

### 3.4 Monoaminergic outputs were more explainable than serotonin modulation terms

To quantify the contributions of the 14 predictors to the regression-predicted variables, the LMG and Pratt indices were used (Draper and Smith, 1998; Kutner et al., 2005). Because R^2^ increases monotonically with the number of predictors, this study also evaluated adjusted R^2^, which penalizes unnecessary variables.

Table 7 summarizes the adjusted R^2^ values for arousal, valence, NE, DA, 5-HT, *x*_1_, and *x*_2_. Although arousal showed R^2^ ≥ 0.9 for all participants and valence showed R^2^ ≥ 0.9 for seven participants, adjusted R^2^ revealed that high goodness-of-fit was limited to a subset of participants. Variable importance was interpreted only for regression models exhibiting non-negative adjusted R^2^ values. Because negative adjusted R^2^ indicates that the regression model is statistically inappropriate, variable-importance indices (LMG and Pratt) derived from these models were not interpreted. For arousal, Participants No. 26, No. 31, and No. 40 showed adjusted R^2^ ≥ 0.6; for valence, Participants No. 26, No. 31, No. 38, and No. 40 did so.

**TABLE 7:** Adjusted *R*^2^ values of the 14-variable regressions for arousal, valence, norepinephrine (NE), dopamine (DA), serotonin (5-HT), *x*_1_, and *x*_2_ (for Participant No. 26, No. 31, No. 38, and No. 40 among nine participants and for the pooled data across all nine participants).

Participant no.	Arousal	Valence	NE	DA	5-HT	x_1_	x_2_
26	**0.948**	**0.922**	0.457	**0.983**	**0.999**	**0.637**	**0.913**
31	**0.609**	**0.904**	**0.874**	**0.975**	**0.904**	0.513	**0.898**
38	0.465	**0.621**	**0.999**	**0.922**	**0.992**	0.517	**0.630**
40	**0.835**	**0.757**	**0.663**	**0.999**	**0.999**	**0.936**	**0.771**
All	0.041	0.0	0.195	**0.984**	**0.700**	**0.650**	0.130

Cases with adjusted *R*^2^ ≥ 0.6 are shown in bold.

A clearer distinction emerged when the five latent parameters were analyzed separately. Adjusted R^2^ exceeded 0.6 for DA and 5-HT in most cases, and for NE in all but two participants, indicating that the monoaminergic outputs were relatively well-explained by the measured physiological variables. In contrast, *x*_1_ and *x*_2_ showed high adjusted R^2^ only in a limited subset of participants, indicating that the serotonin modulation terms were less readily explained by the measured variables. Notably, participants with higher adjusted R^2^ for *x*_1_ and *x*_2_ also tended to show higher adjusted R^2^ for arousal and valence (Table 7).

Figure 8 shows the LMG and Pratt contributions for arousal and valence in Participants No. 26, No. 31, and No. 40, who exhibited high goodness-of-fit for both dimensions. Results for Participants No. 25, No. 37, and No. 38 are provided in Supplementary Figure 4. For Participant No. 26, the largest contributions to arousal came from PFC/PAC α-band power, pO_2_, and PFC δ- and θ-band power, whereas the largest contributions to valence came from PFC δ-band power, PFC/PAC α-band power, and PFC θ-band power. For Participant No. 31, arousal was influenced most strongly by PFC γ- and β-band power and OCC α-band power, whereas valence was dominated by pCO_2_. For Participant No. 40, arousal was influenced most strongly by MAX_SBP, pO_2_, and pCO_2_, whereas valence was influenced most strongly by PFC β-band power and OCC α-band power. Negative Pratt coefficients indicated that some predictors, such as PFC θ-band power in Participant No. 26 or OCC α-band power in Participant No. 40, contributed in the opposite direction to the predicted affective dimension.

**FIGURE 8:**
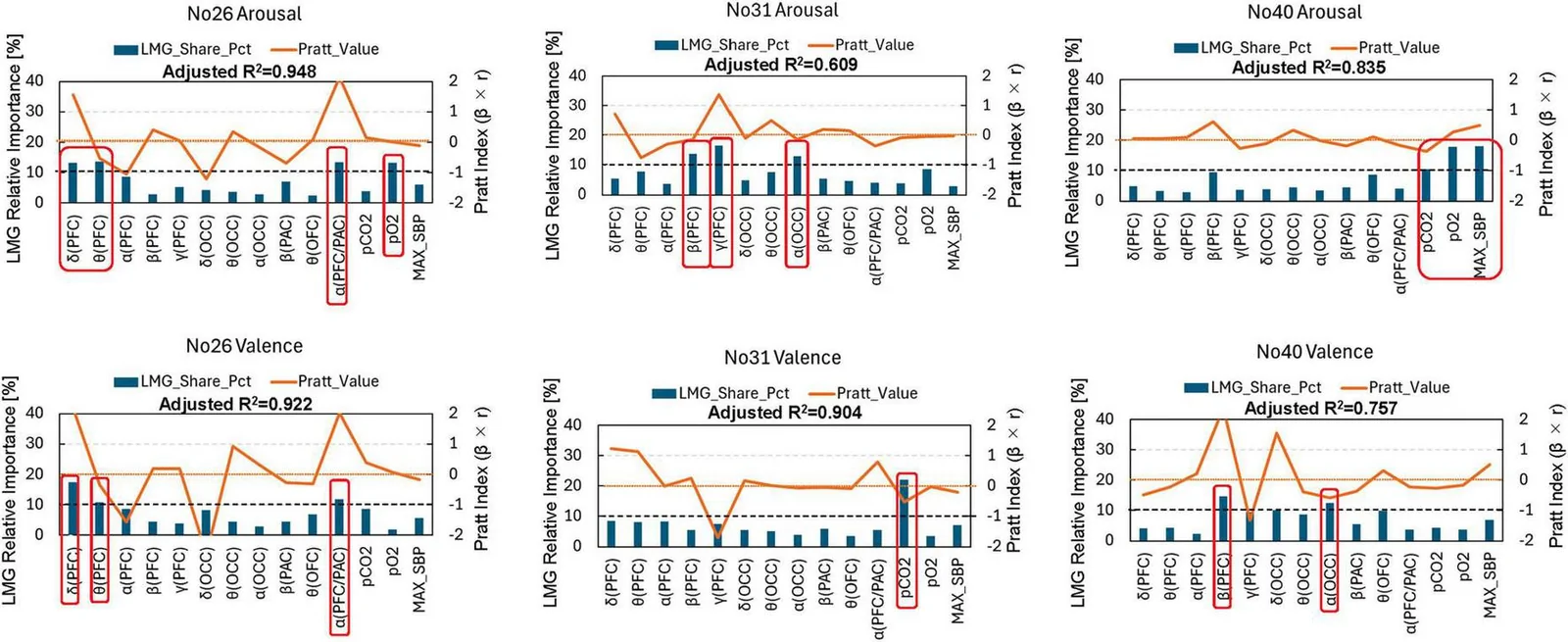
Predictor contribution ratios based on the Lindeman, Merenda, and Gold (LMG) index and Pratt index for arousal and valence, together with adjusted *R*^2^, for the 14 variables (Participants No. 26, No. 31, and No. 40).

Across all participants and stimulus conditions, the predicted values of pCO_2_, pO_2_, and MAX_SBP varied within narrow ranges (−4.7 to 6.5, 25.5 to 27.0, and −3.3 to 2.9 mmHg, respectively) relative to their pre-stimulus baseline maxima. Thus, although these cardiorespiratory variables contributed meaningfully to the regression analyses, their absolute variations across participants and stimuli were modest.

## 4 Discussion

### 4.1 Main findings and significance of the framework

The present study introduces a subject-specific mechanistic framework linking EEG-derived cortical activity to subjective affect through a coupled deep-brain–cardiorespiratory system. Under the present calibration procedure, five latent parameters—NE, DA, 5-HT, and two serotonin modulation coefficients (*x*_1_ and *x*_2_)—were sufficient to reproduce individual valence–arousal ratings across 144 participant–stimulus instances in the AMIGOS dataset (Miranda-Correa et al., 2021; Figure 3 and Supplementary Figure 1). Importantly, the value of this framework does not primarily lie in general-purpose affect classification but in its ability to represent affective ratings using a compact and interpretable set of physiological and modulation-related variables. In this sense, the model provides a hypothesis-generating account of how cortical activity, bodily feedback, and monoaminergic dynamics may jointly contribute to subjective affect. The proposed mapping between EEG spectral features and deep-brain nuclei should not be interpreted as a direct physiological correspondence. Instead, these mappings were introduced as biologically informed hypotheses derived from converging evidence across electrophysiological, neuroimaging, and neurophysiological studies. Their primary purpose is to provide an interpretable computational framework, which should be validated in future studies using simultaneous EEG-fMRI or invasive neural recordings. Although *x*_1_ and *x*_2_ were estimated through model fitting in the present study, future work should investigate whether these latent parameters can be predicted from independently measurable characteristics, such as personality traits, baseline autonomic physiology, resting-state EEG, or stimulus-related features. Such analyses would provide stronger evidence for the physiological validity of these latent variables and further improve the predictive capability of the proposed framework. A distinctive feature of the present framework is that cardiorespiratory variables are not treated merely as additional statistical predictors of affect but are incorporated into a dynamical system linking peripheral physiological states with hypothesized neuromodulatory processes (Critchley and Harrison, 2013). This coupling provides a computational representation of how cortical and bodily physiological signals may jointly shape individual affective states.

### 4.2 Within-participant consistency and cross-participant variability

The leave-one-stimulus-out cross-validation analysis provided a more stringent assessment of within-participant predictive validity than the goodness-of-fit analyses. Across all nine participants, the mean RMSE was 0.380 ± 0.288 for arousal and 1.283 ± 1.512 for valence (Figure 5 and Table 6). Thus, prediction of unseen stimuli was more consistent for arousal than for valence, while substantial inter-individual variability was observed, particularly for valence. One possible interpretation of this difference is that arousal is more directly coupled to measurable physiological activation, including autonomic and cardiorespiratory responses, whereas valence may depend more strongly on subjective appraisal, prior experience, memory, and stimulus-specific context. Because the present framework explicitly incorporates both cortical EEG features and cardiorespiratory variables, its relatively stable prediction of arousal may reflect the stronger physiological constraints on this affective dimension. In contrast, the greater variability in valence prediction may indicate that valence depends on individualized processes that are only partially captured by the physiological variables included in the present model.

The weak cross-participant generalization observed in the regression analyses is also consistent with the substantial inter-individual variability reported for neurophysiological correlates of emotion. Even when individuals are exposed to the same affective stimuli, their cortical, autonomic, and subjective responses may differ because of differences in prior experience, personality, baseline physiological state, and cognitive appraisal (Hamman and Canli, 2004; Stemmler and Wacker, 2010; Siegel et al., 2018). Accordingly, the limited transferability of regression relationships across participants should not necessarily be regarded solely as model failure; rather, it may partly reflect genuine heterogeneity in the physiological organization of affect.

Taken together, the relatively stronger within-participant prediction and weaker cross-participant generalization support the use of subject-specific rather than population-level parameterization in the present framework. This interpretation is also consistent with the variability of the serotonin-related modulation coefficients observed across participants and stimuli. Nevertheless, larger independent datasets will be required to determine the extent to which this variability reflects stable individual characteristics rather than sampling variability or model uncertainty.

### 4.3 Monoaminergic components and serotonin-related modulation

A central result was the clear separation between relatively stable monoaminergic components and more variable serotonin-related modulation terms (Figure 6). Across stimulus conditions, DA and 5-HT changed only modestly, whereas NE showed somewhat greater stimulus sensitivity, and the two serotonin modulation coefficients varied strongly across both participants and stimuli (Figure 7 and Supplementary Figures 2, 3). In addition, regression analyses suggested that NE, DA, and 5-HT were more readily explained by measured EEG and cardiorespiratory variables than *x*_1_ and *x*_2_ (Table 7 and Supplementary Figures 5–9). Within the assumptions of the present model, these findings suggest that the monoaminergic components may reflect a comparatively constrained physiological substrate of affective state, whereas serotonin-related modulation may capture more individualized and context-dependent shaping of valence and arousal. Rather than treating this residual structure as unexplained variance, the present framework suggests that it may represent an important computational signature of subject- and stimulus-dependent affective interpretation.

### 4.4 Relation to other computational models of emotion

The present framework differs from several established computational approaches to emotion in its primary level of description. Active-inference accounts conceptualize affective experience in terms of inference about internal and external states and the regulation of prediction errors, thereby providing a principled framework for linking perception, interoception, and action (Seth and Friston, 2016; Smith et al., 2019). Reinforcement-learning models, in contrast, primarily describe how reward value, prediction errors, and action policies are updated through experience (Moerland et al., 2018). These approaches provide powerful computational accounts of affective cognition and behavior, but peripheral physiological dynamics and specific neuromodulatory circuits are often represented at a relatively abstract level.

The present framework takes a complementary approach. Rather than focusing primarily on belief updating or reward learning, it explicitly links EEG-derived cortical features, cardiorespiratory dynamics, and hypothesized monoaminergic circuits within a single dynamical architecture. In particular, cardiorespiratory variables are incorporated as components of the modeled brain–body interaction rather than simply as statistical covariates. This architecture therefore provides an interpretable intermediate description between measured peripheral/cortical physiology and subjective affective ratings.

The framework also differs from models that represent neuromodulation primarily at an abstract computational level. Here, NE-, DA-, and 5-HT-related components are explicitly associated with hypothesized LC-, VTA-, and RN-related processes, although these associations remain indirect and should not be interpreted as direct measurements of neurotransmitter activity. The principal contribution of the present approach is therefore not to replace active-inference, reinforcement-learning, or other computational theories of emotion, but to provide a complementary physiologically grounded framework for generating testable hypotheses about how cortical activity, cardiorespiratory feedback, and neuromodulatory processes may jointly contribute to individual affective states.

### 4.5 Relation to psychological constructionism

This distinction between relatively constrained physiological components and more individualized modulation terms is broadly compatible with theoretical accounts that distinguish basic affective state from higher-order contextual interpretation. In particular, psychological constructionist accounts propose that affective experience emerges from both core affect, grounded in interoceptive bodily states, and subsequent categorization or interpretation shaped by context, memory, and conceptual knowledge (Barrett, 2017). In the present framework, the pre-adjustment monoaminergic state—determined by EEG-derived cortical drive together with cardiorespiratory feedback variables such as pCO_2_, pO_2_, and MAX_SBP—may be interpreted as a model-based correlate of core affect. By contrast, the serotonin-related modulation terms *x*_1_ and *x*_2_, which strongly influence the final position of the affective state on the circumplex and vary substantially across participants and stimuli, may reflect a more context-sensitive transformation of this underlying state. Although this interpretation remains hypothetical, it provides a useful conceptual framework for understanding why subjective affective ratings can differ markedly even when external stimulus conditions are shared.

### 4.6 Serotonin modulation and expansion across affective space

The interpretations in section “4.3 Monoaminergic components and serotonin-related modulation” are also consistent with the observation that serotonin exerts complex and sometimes bidirectional effects on arousal, sleep, punishment sensitivity, and behavioral regulation (Blier, 2001; Harkin et al., 2025; Monti, 2011; Rogers, 2010). In the present model, this uncertainty was represented by allowing the serotonin modulation coefficients *x*_1_ and *x*_2_ to take either positive or negative values within bounded ranges. This choice was not only physiologically motivated but also computationally informative: without serotonin adjustment, the predicted affective states were largely confined to the unpleasant region of the circumplex, whereas inclusion of *x*_1_ and *x*_2_ expanded the distribution across all quadrants. Within the assumptions of the present model, this result suggests that serotonin-related modulation may provide a computational mechanism for transforming a relatively restricted physiological affective state into a broader range of subjectively experienced valence–arousal configurations. In this sense, the model supports the possibility that serotonin-dependent modulation contributes not simply to affective intensity but to the qualitative organization of affective space.

### 4.7 Implications for anxiety- and depression-related affective states

Our observation may also be relevant to the interpretation of altered serotonergic function in anxiety- and depression-related affective states. In the circumplex model, anxiety and distress are typically located in the unpleasant/high-arousal region, whereas depressive states are more closely associated with unpleasant/low-arousal configurations (Russell, 1980). Reduced or dysregulated serotonergic function has been linked to threat sensitivity, anxiety-related symptoms, and depressive vulnerability (Lanzenberger et al., 2007; Monti, 2011; Neumeister et al., 2004; Rogers, 2010). In the present model, affective states without serotonin adjustment were concentrated primarily in the second and third quadrants. Within the present model, lower fitted serotonin-related modulation coefficients were associated with a narrower distribution of affective states. This association should not be interpreted as evidence of reduced serotonergic function. These observations may have potential implications for individualized assessment of affective dysregulation. Depression and anxiety are clinically and neurobiologically heterogeneous, with substantial inter-individual variation in the neural circuits associated with affective, cognitive, and self-referential processes (Williams, 2016). Altered autonomic regulation has also been reported in both depression and anxiety, including reduced heart rate variability (Kemp et al., 2010; Chalmers et al., 2014). A framework that represents cortical, cardiorespiratory, and latent neuromodulatory components at the individual level may therefore provide a computational tool for characterizing such heterogeneity. However, the present study was conducted using a non-clinical dataset, and neither monoamine concentrations nor deep-brain activity were directly measured. Accordingly, the fitted serotonin-related coefficients should not be interpreted as biomarkers of serotonergic dysfunction or psychiatric disorders. Future studies involving clinical populations and independent neurophysiological measurements will be necessary before any diagnostic or therapeutic implications can be established.

### 4.8 Limitations and future directions

Several limitations should be considered when interpreting these findings. First, the present analyses were conducted using only the AMIGOS dataset and a relatively small subset of participants with usable EEG data, and the proposed framework should therefore be evaluated on additional independent datasets. Second, the simulations used fixed baseline respiratory and cardiac settings rather than participant-specific physiological baselines, which might have reduced the biological realism of the model. Third, the mappings from EEG band-power features to specific deep-brain structures were based on prior literature but remain indirect and incomplete from a neurophysiological perspective. Although LOSO cross-validation reduced the risk of evaluating the model solely on fitted data, the number of stimulus conditions within each participant remained small. Therefore, the estimated prediction errors may themselves be subject to sampling variability. The relatively large number of predictors compared with the number of within-participant observations also remains an important limitation. Finally, the modeled circuit is necessarily simplified: additional pathways and neuromodulatory systems, including GABA, glutamate, oxytocin, vasopressin, acetylcholine, and endocannabinoids, may also contribute to affective state and its individual variability. Future work should therefore focus on validating the framework in larger and more diverse cohorts, incorporating participant-specific physiological baselines, refining the cortical-to-subcortical mappings, and moving from per-sample calibration of *x*_1_ and *x*_2_ toward predictive estimation of these coefficients from measurable physiological variables and stimulus descriptors.

## 5 Conclusion

This study developed a subject-specific computational framework integrating EEG-derived cortical activity, cardiorespiratory dynamics, and hypothesized neuromodulatory processes to represent individual valence–arousal states. The framework reproduced participant-specific affective ratings using a compact set of latent physiological and modulation-related variables. Leave-one-stimulus-out cross-validation further demonstrated that prediction of unseen stimuli was more consistent for arousal than for valence, while substantial inter-individual variability was observed. These findings highlighted the importance of subject-specific brain–body modeling for understanding individual affective responses.

Future studies should validate the framework in larger and independent cohorts, incorporate participant-specific physiological baselines, and directly evaluate the hypothesized cortical-to-subcortical relationships using multimodal neuroimaging or invasive recordings where feasible. An important further step will be to predict the serotonin-related modulation coefficients from independently measurable variables, including personality traits, baseline physiology, resting-state EEG, and stimulus characteristics. Validation in clinical populations will also be necessary to determine whether subject-specific brain–body modeling can contribute to individualized assessment of affective dysregulation.

## Data Availability

The AMIGOS dataset analyzed in this study is publicly available as described by Miranda-Correa et al. (2021). The source code implementing the deep brain-cardiorespiratory model, EEG feature extraction, and parameter identification procedures, together with scripts for reproducing the main figures and tables, is publicly available on Zenodo at https://doi.org/10.5281/zenodo.19060889.
