# Supplementary material for: A subject-specific mechanistic model of affect links electroencephalography, cardiorespiratory feedback, and monoaminergic dynamics
Source: Front Comput Neurosci. 2026 Sep 18;20:1875657. doi: 10.3389/fncom.2026.1875657 (PMC13631044; doi:10.3389/fncom.2026.1875657)
Supplement: Supplementary file 1 [file Supplementary_file_1.docx]

Supplementary Material

# 1 Supplementary Figures and Tables

**Supplementary Figure 1.** Comparison between model-predicted affect and subjective ratings (Participants No. 09, No. 37, No. 38, No.39, and No. 40). In each plot, the x-axis represents valence and the y-axis represents arousal. Text labels indicate the titles of the 16 movies.

**Supplementary Table 1.** Predicted values of NE, DA, 5-HT, x1, and x2 and the subjective arousal and valence ratings for the 16 video stimuli (Participants No.09 and No.25).

**Supplementary Table 2.** Predicted values of NE, DA, 5-HT, x1, and x2 and the subjective arousal and valence ratings for the 16 video stimuli (Participants No.30 and No.31).

**Supplementary Table 3.** Predicted values of NE, DA, 5-HT, x1, and x2 and the subjective arousal and valence ratings for the 16 video stimuli (Participants No.37 and No.38).

**Supplementary Table 4.** Predicted values of NE, DA, 5-HT, x1, and x2 and the subjective arousal and valence ratings for the 16 video stimuli (Participants No.39 and No.40).

**Supplementary Figure 2.** Computed quantities of the three neurotransmitters and the two 5-HT adjustment terms across the 16 video stimulus conditions (Participants No.09, No.25, and No.30).

**Supplementary Figure 3.** Computed quantities of the three neurotransmitters and the two 5-HT adjustment terms across the 16 video stimulus conditions (Participants No.38, No.39, and No.40).

**Supplementary Figure 4.** Predictor contribution ratios based on the LMG and Pratt indices for arousal and valence, together with adjusted , for the 14 variables (Participants No.25, No.37, and No.38).

**Supplementary Figure 5.** Predictor contribution ratios based on the LMG and Pratt indices for NE, DA, and 5-HT, together with adjusted R², for the 14 variables (Participants No.09, No.25, and No.26).

**Supplementary Figure 6.** Predictor contribution ratios based on the LMG and Pratt indices for NE, DA, and 5-HT, together with adjusted R², for the 14 variables (Participants No.30, No.31, and No.37).

**Supplementary Figure 7.** Predictor contribution ratios based on the LMG and Pratt indices for NE, DA, and 5-HT, together with adjusted R², for the 14 variables (Participants No.38, No.39, and No.40).

**Supplementary Figure 8.** Predictor contribution ratios based on the LMG and Pratt indices for x1 and x2, together with adjusted R², for the 14 variables (Participants No.26 and No.31).

**Supplementary Figure 9.** Predictor contribution ratios based on the LMG and Pratt indices for x1 and x2, together with adjusted R², for the 14 variables (Participants No.38 and No.40).
